# The worldwide patent landscape of dental implant technology

**DOI:** 10.1186/s40824-022-00307-0

**Published:** 2022-10-23

**Authors:** Woo Jin Kim, Young-Dan Cho, Young Ku, Hyun-Mo Ryoo

**Affiliations:** 1 Department of Molecular Genetics & Dental Pharmacology, School of Dentistry and Dental Research Institute, Seoul National University, 1 Gwanak-ro, Gwanak-gu, Seoul, 08826 Korea; 2 Technology Management Office, R&DB Foundation, Seoul National University, Seoul, Korea; 3 Department of Periodontology, School of Dentistry and Dental Research Institute, Seoul National University and Seoul National University Dental Hospital, Seoul, Korea

**Keywords:** Dental Implant, Patent landscape, Implant Materials, Implant Technology

## Abstract

In an aging society, quality of life improvement is emerging as an important issue, and as implants are accepted as the core of oral rehabilitation treatment, competition for leadership in developing related technologies is intensifying. In this trend, unlike what is evident in the literature, the patent landscape shows the status of industrial-based technology development. A database analysis of a total of 32,237 dental implant patents shows improvements in technology, diverse geographical characteristics, and new advances toward technological convergence in this field. Technologically, dental implant technology has shown a tendency to develop from conventional implant materials and surface treatment technologies to new material technologies making use of substances such as pure zirconium and tantalum or software technologies related to diagnosis and prognosis. Regionally, dental implant technology, which was developed mainly in Europe and the Unites States in the past, is growing explosively in East Asian countries accompanied by the recent growth of the Asian market. In summary, dental implant technology seems to be developing while trying to converge with various technological areas based on the local market environment. Therefore, it is necessary to develop a new dental implant material technology that is highly applicable to the development of hybrid information/communication technology and is suitable for a new manufacturing method. Our study may provide important information to help basic and translational researchers and their financial supporters set their research directions in advancing the development of dental implants.

## Introduction

Improvement in quality of life in an aging society is emerging as an important issue, and accordingly, the market for oral rehabilitation treatment is rapidly expanding [1]. As dental implants are accepted as the core of oral rehabilitation treatment, the competition for leadership in the development of related technologies is intensifying [2]. In the past, improving the success rate of implant treatment was the main goal of technological development, but recently, diversified technology development patterns have emerged, and in particular, convergence with other technologies is actively taking place. In addition, it seems that this technological change is the basis for the regional impact due to the rapid expansion of the dental implant market.

The global population is aging rapidly, and the growth potential of the dental implant market is gradually increasing. According to the World Population Aging report, the world’s older population continues to grow at an unprecedented rate [3]. There were approximately 703 million people aged 65 years or above in 2019, and the number of elderly people is expected to reach 1.5 billion by 2050 [4]. The increase in the elderly population accompanies an increase in the number of patients suffering from dental diseases.

Historically, since Swedish orthopedic surgeon Dr. Branemark applied dental implants in patients for the first time in the mid-1960s and reported the fusion of titanium and bone (called osseointegration), basic techniques for dental implant treatment have intensively developed in the United States of America (USA) and Europe [5]. Accordingly, the implant market initially grew mainly in America and Europe and became an important business field of global medical device companies, such as Nobel Biocare, Danaher, Zimmer, Straumann and Dentsply. Major technologies for dental implants overlap with those for orthopedic implants, and orthopedic medical device companies, such as Medtronics, are also actively participating in technology development.

While the North American influence is still dominant in the market, the Asia Pacific (APAC) region is expected to show the highest growth rate due to its increasing healthcare expenditures. The global dental implant market recorded 5% growth in 2020 and was predicted to present a compound annual growth rate (CAGR) of 5.0% through 2026. Regionally, the dental implant market in the APAC is expected to grow 1.6-fold faster than those in other regions by 2027, with a CAGR of 8.1%, because the number of people aged 65 years and older is expected to triple between 2010 and 2050, reaching up to more than 1.3 billion people [3]. In particular, the growth of the Chinese market is pronounced, exceeding USD 0.7 billion in 2020 (19.4% of the global market), with an expected CAGR of 28% [6].

Titanium implants account for almost all dental implant technologies and for the greatest portion of dental implant patents, reaching up to 91%. Major technical concerns of titanium implants are increased osseointegration and structural stability. From the first endosseous implant patent (US943113A) reported in 1909, the design of the implant fixture has evolved into a root-form type (representative, US5015186A). Additionally, technologies that modify the implant surface with unique tomography through mechanical, chemical, electrochemical or laser treatments to promote accelerated healing and osseointegration have been developed (representative, JP2003199471A). Other implant materials, including zirconium and alloys made by mixing titanium with other metals, have been developed (representative, US13470761A).

**Fig. 1:**
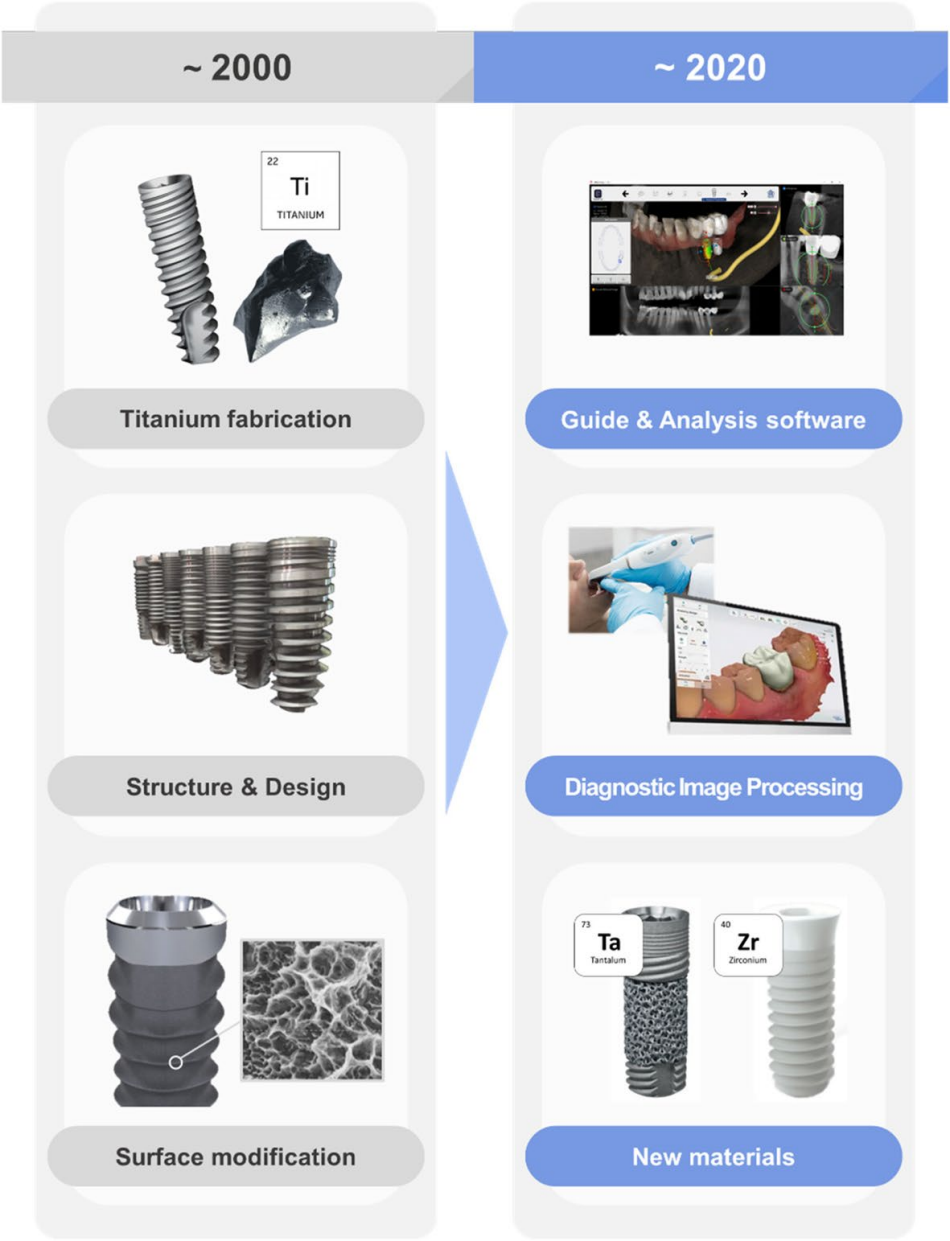
Trends of technological changes in dental implant patents. In the early 2000s (2000 to 2004), dental implant technology focused on the development of manufacturing methods, structures and surface treatment technologies, but in recent years (2016 to 2020), the development of guides, modeling methods, and communication technologies used for treatment and diagnosis through the convergence of IT and CT has emerged prominently. Additionally, tantalum and zirconium are being studied as alternatives to titanium

However, over the last two decades, the major focus of dental implant technology has been on innovations to improve accuracy and convenience through convergence with information technology (IT) and communication technology (CT). Tools for technological crosslinking that use computational algorithms or database analysis for assisting in treatment and diagnosis, such as surgical guides (representative, KR201710954A) or diagnostic image analysis (representative, GB200514554A), are also being adopted, thereby driving the overall technology development. In addition, new materials, such as pure zirconium and tantalum that is not mixed with titanium, are emerging as alternatives to titanium. (Fig. 1).

In this review, we observed the changes in technology development trends of dental implants over the last 20 years through patent database analysis and, in particular, analyzed the ideal directions of technology development in response to changes in the dental implant market.

### Analytical background

For more precise patent data collection, we adopted the search criteria from a previous report [7] and supervised patent law professionals to collect optimal global patent documents related to dental implants. We collected registered or pending patents from 60 countries from 2000 to 2020 to extract patents that were active in the same period based on US patent law. In most countries, including the USA, 20 years after filing is regarded as the duration of a patent. Additionally, this study retrieved patents with priority filing dates before 31 December 2020 because a patent filed in one country is not published until 1 year and 6 months before the priority filing date (usually called the “dark period”).

The Derwent innovation patent database (https://clarivate.com/products/derwent-innovation/) was used to extract patents related to dental implants [8]. The term “dental implant”, the term for each constitutive part, and international patent classification (IPC) codes were used for the patent search. Each term was retrieved and collected, with data covering items including the title, abstract and claims. The detailed search query of each term is shown in Table 1 and search result of each database categories is shown in Table 2.

**Table 1:** Analytic patent database categories and queries

Category	Keywords	Query
Dental Implant	Dental implant and IPC code A61C (Dentistry; Apparatus or Methods for oral or dental hygiene)	(dental^*^ ADJ implant^*^) AND IPC = ((A61C^*^));
Fixture	Dental implant fixture and IPC code A61C	(dental^*^ ADJ implant^*^) AND (fixture^*^ OR fixture^*^ OR fix^*^ OR screw^*^) AND IPC = (A61C^*^);
Abuitment	Dental implant abutment (connecting element) and IPC code A61C	(dental^*^ ADJ implant^*^) AND (abutment^*^ OR connect^*^ OR connector^*^) AND IPC = (A61C^*^);
Artificial Teeth	Dental implant artificial teeth and IPC code A61C	(dental^*^ ADJ implant^*^) AND ((artificial^*^ ADJ (tooth^*^ or teeth^*^)) or (cap^*^)) AND IPC = (A61C^*^);

Search Periods (Year): 2000 ~ 2020

Search date: March. 2. 2021

**Table 2:** Search results of dental implant patents published between 2000 and 2020

Category	Results			
	**Total**	**DWPI**	**INPADOC**	**Applicants**
Dental Implant	32,237	10,478	10,505	22,360
Fixture	13,942	5,316	5,403	10,534
Abutment	16,387	6,802	6,677	12,539
Artificial teeth	2,304	1,140	1,172	1,811

After data collection, patent professionals manually excluded irrelevant or duplicate patents. The patent data collected were analyzed to retrieve various information from the bibliography, such as the applicant, inventor, application year, application country and IPC code. Moreover, to assess the technical evaluation, we used text mining-based patent clustering to describe the regional technical development directions and characteristics by employing the software platform ThemeScape Map.

## Results

### The recent trend of patents related to dental implants

According to the patent analysis, the direction of technology development in the field of dental implants over the past 20 years was represented by "technological convergence" and "localization".

**Fig. 2:**
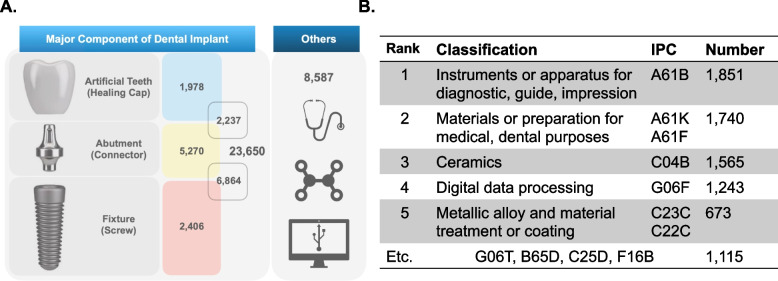
Current state of dental implant patents. **A** The result of patent technology classification by implant part, showing that the patents applied for after 2000 do not concentrate on the implant itself. **B** The number of patents by IPC for technologies included in the “others” category

The technology field of the recently published dental implant patents shows a tendency to deviate from the technology for the implant itself. Among a total of 32,237 patents, there were 23,650 patents (73.4%) for implant body parts (indicated by “fixture”, “abutment” and “artificial tooth”) and 8,587 patents (26.6%) not directly related to the implant body (indicated by “others”). (Fig. 2A).

Patents in the “others” area included technologies for accurate diagnosis and surgical assistance during dental implant procedures, as well as new material technologies. In the IPC analysis of 8,587 patents, 21.5% (1,851 cases) and 20.3% (1,740 cases) were in the A61B category, represented by "diagnosis, guide and impression-taking related technology", and the A61K and A61F categories, corresponding to "surgical instruments and auxiliary devices", respectively. The C04B and C22C category, which includes "tantalum and zirconia" as a new material technology, represented 18.2% (1,565 cases) and corresponded to the third most common patents found in the “others” field. Interestingly, the G06F category, which includes "image processing technology, data mining, and artificial intelligence (AI) technology", represented a high percentage (14.5%, 1,565 cases). (Fig. 2B).

### Patent network analysis using the citation index

The citation index of a patent is an important measure that allows us to infer how important a patent is in a particular technical field [9]. During the prosecution of a patent application, an examiner will look for prior art related to the novelty, obviousness or inventive step associated with an invention.

**Fig. 3:**
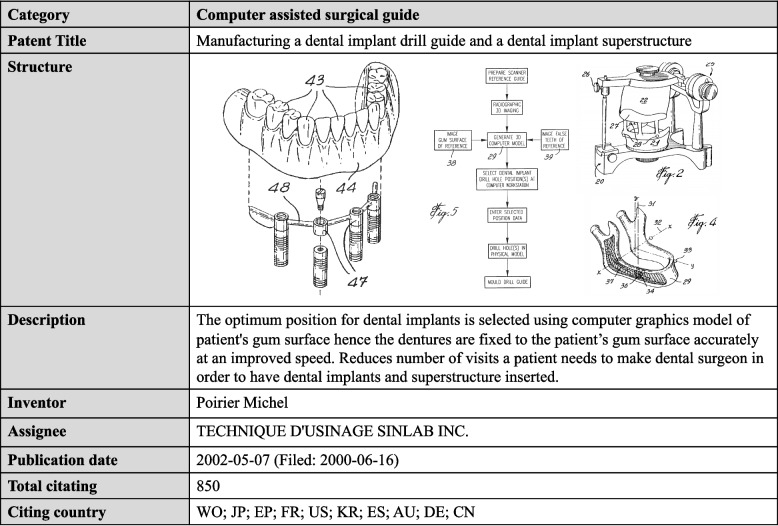
The most cited patent in the dental implant field. The most cited patent filed between 2000 and 2020 is on the technique of the optimal positioning guide for implant-assisted edentulous denture treatment

The patent most frequently cited in the dental implant field for the past 20 years was "manufacturing a dental implant drill guide and a dental implant superstructure (US6382975B1)", which was cited a total of 558 times in patents worldwide, including patents in Japan, Europe, the USA, Korea, France, Spain, Australia, Germany and China. This patent described an optimum positioning guide for dental implant surgeries selected from computer graphic models of the patient's gum surface to improve denture fitting; its IPC is A61C1/084 (“positioning of implant drill guides”) and A61C9/0053 (“optical means or methods”, e.g., scanning the teeth by a laser or light beam). (Fig. 3).

Considering that the number of citations of other patents was less than ten, on average (mean = 8.91 times), the significant number of citations of the patent noted above indicates that it has served a basis for many inventions and that patents have been distributed considerably in the field of dental implants. Collectively, in the past 20 years, dental implant patents have tended to expand into new areas rather than focusing on the implant itself.

### Recent advances in surface treatment technology

The characteristics of dental implants, such as the structure, material and fixture surface characteristics, are important factors that influence the osseointegration of dental implants and have been the center of technological development. Among them, increased surface area and bioactivity through surface modification are important determinants of short- and long-term clinical success rates. Therefore, developing various methods to increase the surface energy and roughness of the fixture have been important technical areas of focus in the dental implant field.

Early in the development of dental implants, most fixtures had machined surfaces, with lower surface activity and unpredicted osseointegration success rates. To date, various surface treatment methods have been attempted to improve these shortcomings. Before the 2000s, the basic technologies mainly used for dental implant manufacturing and crucial to implant surface treatment, such as plasma spraying, blasting, etching and oxidation techniques, had already been developed.

IPCs A61C8/0013, A61C8/0015 and A61L2430/02 are characterized by “implant tools by material or composition, e.g., a ceramic, surface layer, metal alloy with a surface layer, and coating” and “modification of an implant surface to improve biocompatibility, cell growth, and biomolecule fixation, e.g., plasma treatment” and constitute a field in which much academic research has been conducted. In the last decade, a new type of surface treatment technology has emerged using the principles of the basic technologies mentioned above. New progressions appeared in remarkably diverse directions, but broadly, they appeared in the direction of imparting nano/microtopologies or bioactivity through basic surface treatment methods. As an example, titanium oxide plasma spraying (US6464889B1) and tantalum/strontium (CN106215237A) or hydroxyapatite/calcium phosphate coating (US20050221259A1) increased the surface roughness and improved the surface chemistry compared to those of conventional fixtures. Additionally, recently, the convergence of different technologies has emerged as a new technological development trend. WO2012110816A1 suggested the plasma spraying of a two-layered coating, with titanium dioxide or zirconium dioxide forming the initial layer and hydroxyapatite forming the subsequent layer. Additionally, US9421151B2 introduced gradient coatings of titanium-hydroxyapatite fabricated by low-pressure plasma spraying. Some techniques have overturned the common conception of the traditional implant fixture structure where the treatment is limited to the thin surface layer. As introduced in US16489508A, a thin structural portion of titanium is coated with thicker layers of 3D-printed ceramic or polymer components to enhance osseointegration.

IPCs A61C8/0015, A61F2002/30925 and A61F2002/30925 are characterized by “implant tools characterized by material or composition, e.g., a coating as a conversion layer or an oxide layer” and “special external or bone-contacting surfaces, e.g., coatings for improving bone ingrowth fabricated by shot-, sand- or grit-blasting and etching”. This field of technology focuses on topographical changes of the implant surface rather than the coating. Early implants were produced by machining and had a smooth surface that was unsuitable for osseointegration [10]. Many mechanical and chemical methods have been attempted to obtain sufficient surface roughness. Representatively, sandblasting, large grit and acid etching (SLA) induces a change in the topography of the implant surface by sandblasting with 0.25–0.5-mm-long grit corundum followed by acid etching, as introduced in US20040210309A1.

Independent of the technological categorization above, many patents applied for from 2000 to 2010 had to do with coating the implant surface with a bioactive or bioderived organic material. Growth factors (bone morphogenetic protein, platelet-derived growth factor, transforming growth factor beta, fibroblast growth factor, vascular endothelial growth factor, etc.), peptides, and extracellular matrix components (collagen, chondroitin sulfate, vitronectin, hyaluronic acid, etc.) were applied to increase the physiological modification of the implant surface. However, there has been a decline in such patent application attempts since 2013.

### Recent advances in dental implant fixture materials

The oldest recorded dental implant patent (1909, US943113A) describes the use of a noncorrosive material, such as gold, silver, platinum or porcelain, as the fixture material. Additionally, in US2347567A, filed in 1944, a methyl methacrylate polymer was used as a material for the fixture. Patents (US2857670A, US3386169A) published between the 1950s and 1960s also used metal materials, such as stainless steel or cobalt/chromium alloys. However, in the 1960s, implants using titanium were featured in a several patents including US3579831A. The first records of zirconium alloys used in dental implants were filed in 1974 (US4040129A).

Titanium and its alloys have favorable advantages as dental implant fixture materials, including osseointegration, biocompatibility, mechanical properties and corrosion resistance [11]. As a dental implant material, titanium is the "gold standard", and 91.4% of patents filed in the last 20 years are based on titanium and its alloys. However, recently, the developing dental technology is fueled by the advent of various materials meant for dental implant. In patents submitted from 2000 to 2010, the proportion of implants with titanium and its alloys as base materials was 95.5%, but in patents filed from 2010 to 2020, the proportion decreased to 87.3%.

The most patented material for these nontitanium implants is zirconia ceramic. Since the first full zirconia implant patent was applied for in the USA in 1975 (US54602375A), many such patents have been filed, mainly in Japan and Europe. Despite the various clinical limitations (poor physical properties, fracture incidence, osseointegration and clinical complications) revealed in previous studies [12], the development of zirconia for use in full implants continues.

Although there are various methods for improving the properties of zirconia, dental implant patents mainly focus on three methods, represented by yttrium-stabilized tetragonal zirconia (3Y-TZP), alumina-toughened zirconia (ATZ) and zirconia-toughened alumina (ZTA).

Yttrium oxide has been used for a long time to strengthen ceramics, and a patent that proposed the usage of magnesium, calcium, lanthanide or yttrium oxide in dental implants was applied for in Japan in 1984 (JP61063568A). Then, implants were developed in which yttrium was applied along with tetragonal crystalline zirconia, and attempts were made to enhance the properties of zirconia ceramics by adding bismuth, terbium, erbium or manganese oxide (EP2013195607A). However, recently, there have been many patents proposing the usage of 3Y-TZP as a material for immediate or processed artificial teeth rather than one for implant fixtures (KR2251422B1).

ZTA and ATZ also have a long history as reinforced ceramics. A ceramic/crystallized glass composite inorganic biodental implant disclosed in JP198746476A in 1987 was composed of a ZTA ceramic dispersed in crystallized glass. In order to compensate for the insufficient strength of ceramics while preserving biocompatibility, dental implant fixtures made of titanium in the part in contact with the bone and ZTA in the part in contact with the soft tissue, were also developed (JP2121654A).

Recently, the fusion of various materials has been performed to improve the physical properties of ceramic fixtures. Zirconia, alumina, and yttrium have been used in one mixture, and the application of new materials such as graphene oxide or tantalum oxide is also being attempted (e.g., CN110885248B).

### Recent advances in dental implant structures

The characteristics of dental implant structures, such as the fixture geometry (parallel-walled, conical or root form (hybrid)), thread structure and connector design, are important factors that influence the osseointegration of dental implants and have been the center of technological development.

Since the concept of direct contact between the bone and the surface of a dental implant was mentioned in 1939, osseointegration has been the key to the success of treatment with dental implants since the 1960s [13]. Therefore, the design of dental implants was developed considering the balance between the increase in the contact area with the surrounding bone, the loading stress distribution and the convenience of the operation. Thus, the primary categories in the direction of dental implant design and development are shape, length, diameter, thread shape, spacing, and additional design elements, and the secondary categories are the connecting structure and type of prosthesis.

Primary categories, including the fixture design of dental implants, account for a notable proportion of patents prior to 2000, but from 2000 to 2020, the proportion of patents related to secondary categories increased. The shape of dental implants, which can influence implant biomechanics, was one of the most controversial design aspects in early dental implant patents. The initial implant shapes were developed in parallel with various forms, such as cylinder, screw, nail-cone, bone cage, blade and subperiosteal designs, but after the 1990s, the shape was unified into a screw type that promotes osseointegration and initial retention. From the 1990s to the mid-2000s, the fixture geometry of screw-type implants was the mainstay of patent development. In the early 1990s, many screw-type dental implants were in the form of parallel-walled cylinders (e.g., US4960381A). Then, the first conical implant patent was applied for in France in 1990 (FR2636832A1). From then to the 2010s, patents related to the shape of parallel-walled and conical implants were applied for a similar rate. Since 2010, a combination of the parallel-walled and conical geometry, called a root shape or hybrid, has been the dominant geometry in dental implant patent applications.

Thread design is an important technical field in terms of the stress distribution and contact area. From the initial patent of the dental implant screw, the threads were mainly self-tapping, and the cutting edge was added (e.g., US4863383A). Non-self-tapping threads, which were also developed for compressive stress acting on the surrounding bone during initial retention, emerged as an important factor for clinical success (US5007835A). However, as it was shown that self-tapping threads had more advantages than non-self-tapping threads in various studies that continued until the 2010s, self-tapping threads were presently accepted as the norm. Recently, various types of fixture threads have been developed, such as threads of a hybrid design with differences between the crestal and apical levels to reduce postoperative complications (CN107106267B), a drill-less design for improved clinical convenience (US20210128279A1), or a hollow-form design for patient bone sample collection (JP06905338B2).

### Convergence with information/computer technology in dental implant patents

As shown in the patent network analysis, recent implant patents show a tendency to develop through convergence with information technology (IT) and communication technology (CT). The results of text mining analysis of abstracts of patents applied for in the last 20 years could be classified according to the following three key terms: personalized design, guidance and modeling, and data analysis.

The field of personalized design technology is advancing due to the development of new technologies for 3D scanning and printing and computer-aided design/computer-aided manufacturing (CAD/CAM). The need for personalized implants based on the specific condition of alveolar bone has been increasing, and a patent for a polymer material-based shell manufacturing process using digital impression data was applied for as the first customized implant patent in 2001 (US6821462B2). At the same time, a patent for manufacturing artificial teeth (or other types of restorations (e.g., restoration core or assembly)) for dental implants by processing preshaped block ceramics using CAD/CAM was also published. Afterward, the number of patents related to personalized design increased rapidly from 2005, reaching a maximum in 2014. Representatively, there is a patent for a zirconia fixture created using CAD/CAM (KR2009009407A), a customized connection part (JP2007222225A), and CAD/CAM image processing for precision machining (US8897526B2). The number of patents involving 3D printing increased after 2013. The patent for using 3D printing directly on the implant body was applied for in 2014 and discloses a nozzle of a 3D printer designed to print ceramics (KR2015085211A) and filament resin, including ceramic filler and binder resin composite, for 3D printing of the implant body (KR2016059302A). However, there are many patents for 3D printing technologies that are used in parallel with diagnostic guides rather than ones for independent implant manufacturing methods. As examples, there is a patent (CN104323865B) for manufacturing orthodontic mini-screws with a 3D printer according to information on a guide plate for orthodontics and a patent for a 3D-printed path guide for use during implant surgery (CN106897574A). Among the patents filed from 2014 ~ 2017, those involving the hybrid technology of CAD/CAM and 3D printing stand out. Patent CN106264762A discloses a dental implant in which a metal/ceramic element is added with a 3D printer to a ceramic input fabricated by CAD/CAM. Recently (2017 ~ 2020), the number of patents using 3D modeling for diagnosis/surgical guidance has increased. Patent US20200078143A1 discloses CAD frame design software for guiding the path of an implant overdenture, and similarly, several patents related to frame design software for a 3D printer have been applied for (e.g., CN112451136A).

Guidance and modeling refer to techniques to increase convenience and accuracy during implant treatment. Traditionally, various impression materials and plaster models have been used to improve the accuracy of prostheses in dental prosthetic treatment. Thus, guidance/modeling technology for setting the precise positional relationship between the prosthesis and the surrounding anatomical structures is an important technology (e.g., US5320529A). However, there are limitations with dental implants in conventional methods because the implant fixture is placed inside the alveolar bone. Therefore, due to the development of medical imaging technologies such as computed tomography (CT) and 3D scanning, a method for predicting the location and path of implants in the alveolar bone before surgery has been developed. Patent KR1797150B1 discloses a method of receiving a tomographic image and determining a person's medial oral profile based on a Y-matching image as an image data processing method for generating an image of a patient's oral profile. Additionally, patent US9730777B2 discloses a CAD/CAM fabrication method using 3D scanning and image data for the fabrication of an implant overdenture.

In the field of dental implants, data analysis technology refers to treatment planning, surgical simulation and oral condition analysis through the evaluation of diagnostic, radiographic and 3D images. With the recent advances in machine learning and artificial intelligence technology, the number of patent applications related to data analysis is increasing. Patent CN111938850A discloses a method for the fabrication of customized artificial teeth based on the gingival shape, thickness and height determined using 3D scanning data. In addition, along with the increase in the number of patents related to robot-assisted implant surgery, a method to support robotic surgery with maxillofacial anatomical modeling through a neural network analysis of radiographic images was developed (RU2019108851A).

### The global landscape of dental implant patent development

**Fig. 4:**
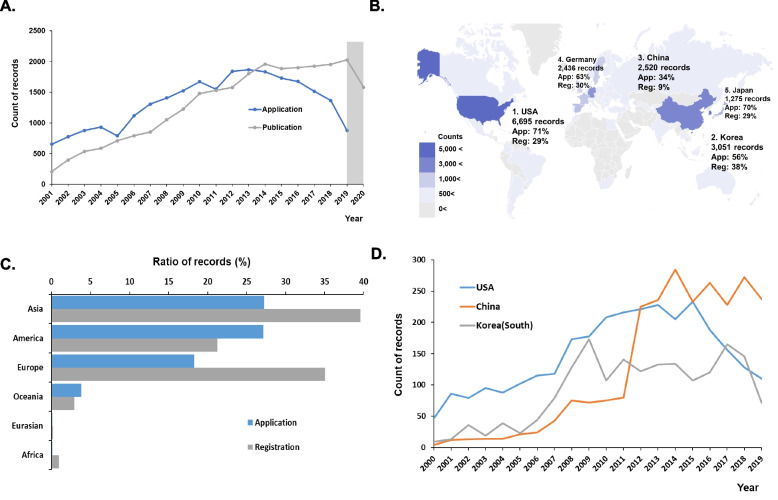
Global technique development trend. **A** The annual trend of global patent applications and registrations for 2000 to 2020. Blue lines indicate number of applications and gray lines indicate published patents per year from 01’ to 20’. Gray box means undisclosed period (dark period). **B** The top 5 global technology development countries: the USA, Korea (south), China, Germany and Japan. App means application, Reg means “registered”. **C** Intercontinental comparison of the international patent entry ratio versus total applications and registrations. **D** Comparison of the number of annual applications in the top 3 countries per year from 00’ to 19’

In the past 20 years, the annual growth rate of the application for and publication of patents related to dental implants has tended to slow down. From 2001 to 2014, the number of dental implant patent applications gradually increased (CAGR (01’ ~ 14’) = 8.26%), but since 2014, the number of patent applications has declined rapidly (CAGR (14’ ~ 19’) = -13.73%). Similarly, the number of published patents has shifted since 2014 (CAGR (01’ ~ 14’) = 18.83%, CAGR (14’ ~ 20’) = -3.48%). (Fig. 4A).

This trend in patent application seems to be related to changes in the major countries represented in the technical field. Prior to 2000, the major countries in the field of dental implant technology were the US, Germany, Sweden and Japan. However, between 2000 and 2020, the major filing countries were the US, Korea (South), China and Germany; in particular, the number of patents in China and Korea increased dramatically. (Fig. 4B).

The intercontinental comparison of international patent entry rates indicates the potential frequncy of interregional technology development. Higher entry rates of applications point to a new or growing market, while lower entry rates can point to low growth or market establishment. Fifty-two percent of worldwide filings are registered, but 48% are pending applications, which indicates protection for active (alive) patents in a region. Overall, 6% of applicants were filed in more than 4 countries. As shown in Fig. 4C, the entry rate in America was still high, while the entry and registration rates in Asian countries were the highest.

The trend of annual patent applications in the top three countries – the USA, China and Korea – changed significantly, the year 2010 serving a turning point. The annual number of patent applications in the USA continued to increase but began to decline around 2014. Interestingly, China did not show a significant increase until 2010 but showed explosive growth after 2011. (Fig. 4D) The development entity of dental implant patents is an important factor underlying these changes. While more than 30% of applicants for patents in the general medical device field originated from research institutions, more than half of the applicants (58%) for dental implant patents were industry based, and only 6% originated from research institutes (university institutions, etc.). In terms of the number of applications per applicant, global medical device companies in the US and Europe were ranked most high, but since 2000, Asian companies (e.g., Chongqing Runze Pharmaceutical, etc.) have applied for dozens of patents each year, showing a high technology development rate. (Table 3).

**Table 3:** Top global patent applicants and their nationalities published between 2000 and 2020

Rnk	Assignee	Country	Patents
1	NOBEL BIOCARE HOLDING AB	Swiss	1364
2	ZIMMER BIOMET INC	USA	1052
3	STRAUMANN HOLDING AG	Swis	948
4	DENTSPLY SIRONA INC	USA	752
5	CHONGQING RUNZE PHARM CO LTD	China	421
6	OSSTEM IMPLANT CO LTD	Korea	319
7	HERAEUS KULZER GMBH & CO KG	Germany	312
8	GC CORPORATION	Japan	256
9	ASTRA TECH AB	Sweden	254
10	DIO CORP	Korea	230
11	WOODWELDING AG	Swiss	218
12	IVOCLAR VIVADENT AG	Liechtenstein	210
13	MEGAGEN IMPLANT CO LTD	Korea	167
14	BIOTECHNOLOGY INST I MAS D SL	Spain	161
15	3 M	USA	127

In summary, the patent trends in the dental implant field after 2000 represent a decrease in the filing rates of formerly major countries in the field and advances in Asia in the field.

### Characteristic regional and temporal technological advances in dental implants

To retrieve regional and temporal information about patent content, we used a large-scale database processing analysis based on text clustering and the ThemeScape thematic text mining tool to discover meaningful trends in technical advances throughout all patent documents.

**Fig. 5:**
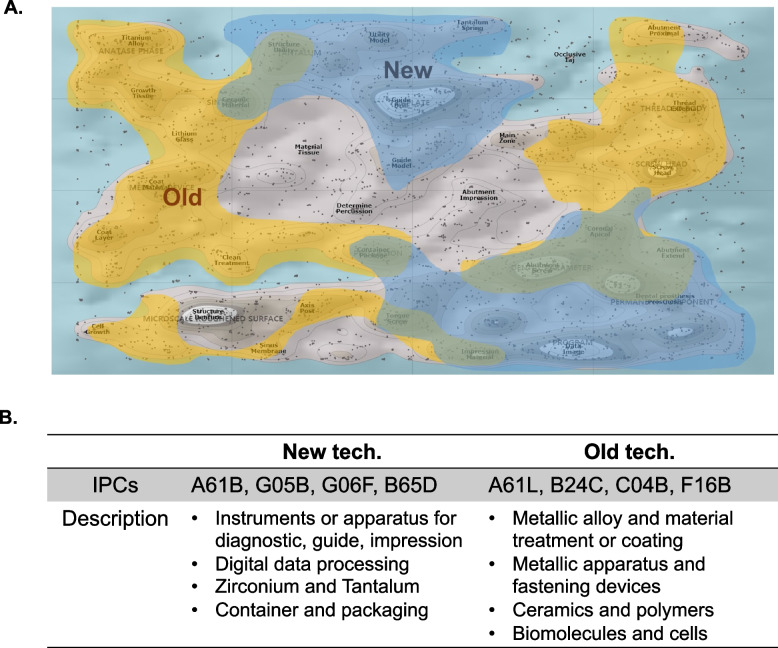
Temporal technical distribution of dental implant patents from 00’ ~ 20’. **A** The ThemeScape-based patent analysis shows that there are differences in applied technology areas by period. Patents filed in the early 2000s (00’ ~ 04’) are marked as “old” (yellow region), and those filed in the last 5 years (16’ ~ 20’) are marked as “new” (blue region). **B** Summary table of technical categories in each period

The patent landscape analysis revealed differences in the technical areas of high patent activity from 2000 to 2020. The text clustering representing the technical areas can be divided into 12 major categories and 32 subcategories that make up each of the major categories. To compare the recent patent areas with those in the early 2000s, we identified the areas covered by patents filed in the first five years (00’ ~ 04’, marked “old”) and the last five years (16’ ~ 20’, marked “new”) in the patent landscape. (Fig. 5A) Interestingly, there was a clear difference between the patents in the new area and the old area. In the old area, mainly metallic material processing and metallic surface treatment (A61F), polymeric and ceramic materials (C04B), surface cleaning technologies (A61K), partial implant structures, fasteners (A61C), and biomolecule-based metallic surface bioactivation technologies (F16B) were mainstream. However, in the new area, mainly diagnostic apparatuses, scanners, surgical guides (A61B), zirconia and tantalum (C22C), diagnostic software, data processing (G06) and packaging technology (B65D) emerged. (Fig. 5B).

**Fig. 6:**
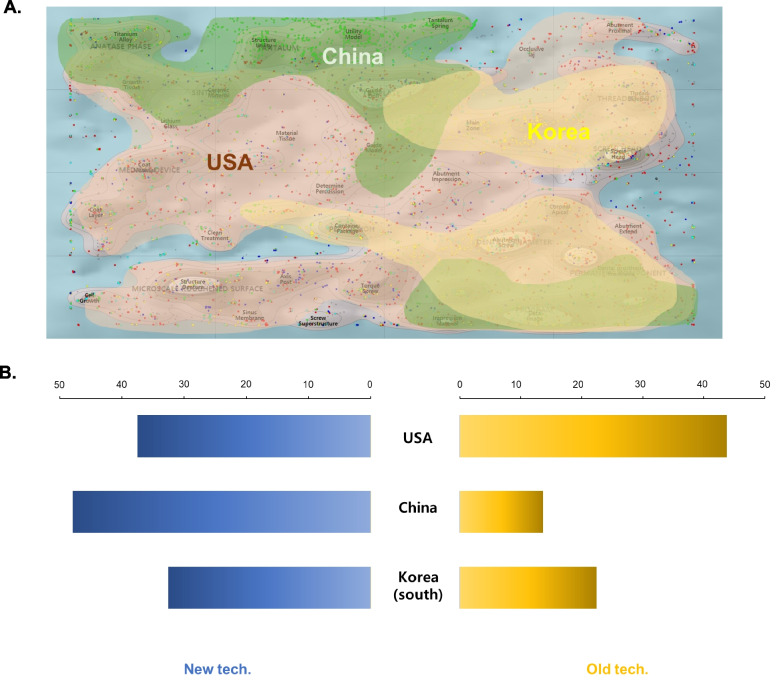
Distribution and ratio of old and new tech in the USA, China and Korea. **A** The ThemeScape-based patent analysis shows that there are differences in the areas of applied technology by country. Patents filed in the USA are indicated in the red region, Korean patents are indicated in yellow, and Chinese patents are shown in the green region. **B** Descriptive diagram of the technical classification of patents recently applied for in these three countries

The technical areas of the top 3 countries leading recent patent applications also showed interesting differences. The USA patents covered all technical areas except for the tantalum segment. However, patents in China focused mainly on the tantalum, surgical guide, and modeling software segments. Korean patents concentrated on the implant component structure and packaging segments. All three countries tried to develop database analysis software and image modeling analysis to assist in precision surgery and guidance. (Fig. 6A).

We analyzed the recent concentration of technologies in the "old" and "new" areas by region. Regionally, a greater proportion of patents in the USA were in the old area (44%) than in the new area (37.5%), while in China, the proportion of patents in the new area was 49%, higher than that in the old area (13%). (Fig. 6B).

## Discussion

Our study has shown the direction of technical advances and the regional contributions of patents in the dental implant technology field that have been filed in the last 20 years through analysis according to technical classification codes (IPCs) and text mining of the patent landscape. The results show convergence with IT as a new direction of technical advancement and the emergence of new materials to complement the limitations of titanium, esthetics and antibacterial capability. This could even represent a paradigm shift, and there are regional groups leading these changes.

The technical classification analysis of recent dental implant patents shows scalability and new attempts in the direction of technology development. Patents can claim materials, structures, methods, functions, or combinations thereof [14], and dental implant patents generally have fixtures, abutments, and artificial teeth as their major components. However, as shown in Fig. 2, approximately 30% of the patents published in the last 20 years have deviated from this generality. These patents in the “others” category span a wide variety of technical areas, except for the aspect of relevance to dental implants, where similarities are difficult to find. Therefore, we classified various and complex "others" patents using the IPC system, and as a result, we observed that many of the recently published patents focused on accessory technologies, especially those for improving the accuracy or convenience of surgery or diagnosis, rather than the dental implant itself. Additionally, we confirmed that many patents are being developed by fusion with IT, such as database or 2D/3D image analysis using software.

The citation index analysis of dental implant patents further supports this trend. As shown in Fig. 3, the patent with the highest number of citations was that for the computer-assisted surgical guide. In addition, "system, method and apparatus for tooth implant planning and tooth implant kits (US20100105011A1)" and informative or assisted technology patents, such as "arrangement and system for the production of dental products and the transmission of information (US6821123B2)" and "method and device for navigation-assisted dental treatment (US6640128B2)", were identified as important patents, with more than 200 citations.

Major dental implant patents have traditionally focused on the materials, structures and modifications (especially surface treatment) of dental implants. While titanium is the standard material for dental implants, patents for implants using ceramic materials such as zirconia and tantalum oxide have recently increased. Zirconia is the most widely developed dental implant material after titanium, but its clinical applications are limited due to its brittleness. Therefore, a patented technology has been developed to enhance its physical properties with yttrium and alumina or to increase its biological activity by applying tantalum oxide. The structure of dental implants has undergone various transformations in the past and is now converging on the root form with self-tapping threads. Technology for the surface treatment of dental implant fixtures is the most studied field for promoting osseointegration and inducing bone formation. While various technologies have been developed, the IPC analysis indicated that surface spraying/coating and surface blasting/etching techniques are the two main technical streams. Surface blasting/etching technology to obtain high surface energy and roughness has converged on the SLA method, but surface spraying/coating technology started with plasma spraying in the past and progressed through the application of bioactive inorganic (hydroxyapatite/calcium) coatings; in recent years, bioactive modified (graphene, tantalum) coatings have been developed. In particular, the development of new manufacturing technologies such as 3D printing provides new opportunities for the development of surface coatings using various materials.

According to the analysis of the development of patented technology [15], the field of dental implant technology has experienced a "revival", and emerging countries are leading the way. The maturity of technology development due to the change in the number of applications and applicants over the past 20 years seems to have declined since 2014. However, during the same period, there were significant changes in the rankings of traditional technology powerhouses and those of emerging countries. In the technological area, data acquisition tools (e.g., 3D scanners) and design/data capturing software technology have led to a new wave of dental implant technology development. Similar to the trend shown in Fig. 4D, the number of patent applications for the main components of dental implants has decreased continuously since 2014. Surprisingly, applications related to personalized treatment and chairside printing, which are considered to be the latest technological fields, are similarly declining. From 2001 to 2020, 3,174 patents related to CAD/CAM or 3D printing in the field of dental implants were found. As a result, the annual average number of applications was 155.2, and the average number of applications since 2016 was 135.4, which is lower than the 10-year average (220.2 cases per year). In contrast, the number of application-related data acquisition tools or design/data capturing software technologies has gradually increased in recent years. Regionally, Asian countries, such as China and Korea, have made remarkable progress, and the number of applications has steadily increased during the same period.

Dental implant technology seems to be facing a new change. As described above, titanium alloys (A61L), implant body structures (A61C), and microscale surface treatments (B24C) have been the traditional major technical areas to prevent infection and promote osseointegration in the field of dental implants. However, applications in these "old" areas are continuously decreasing, and patents in the “new” areas, such as diagnostics (A61B), 3D modeling (G05B), specialized software (G06F) and container or packaging (B65D), are rapidly increasing. This trend in patent applications shows that the technology for the implant itself has been established to some extent, and efforts toward improved accuracy in diagnosis and treatment are increasing. It also seems that technological expansion takes place through convergence with information processing technology.

As described above, it was found that the unique convergence of technological development in the dental implant field over the past 20 years is mainly led by countries emerging in the field. However, these countries show different directions in the development of technology related to dental implant materials. In the field of material technology for dental implants, titanium-based technology has accumulated the highest number of patents [16]. The USA still shows titanium-oriented technological development. However, China is intensively developing tantalum- or zirconium-based material technology independently. For example, the Chinese patent titled "Method for Preparing Medical Porous Metal Material (CN102796891A)" discloses a method of manufacturing a porous tantalum structure using a mixture of tantalum, polyvinyl alcohol, and sodium bicarbonate. In addition, 135 patents, including CN202568504U, disclosed an implant structure that uses tantalum as a major component. The technological direction of using tantalum as a core material for implants is significantly different from the way in which other countries use tantalum (e.g., US patent "Patient-Specific Implants with Improved Osseointegration" (US20110008754A1)), i.e., mainly used for the surface treatment of titanium structures [17]. In addition, tantalum and zirconium, unlike titanium, can be used to manufacture various types of implant fixtures in combination with new manufacturing methods, such as 3D printers or CAD/CAM [18]. The fusion of new materials and new manufacturing methods could present new innovations, such as personalized dental implant fixtures or fixtures that mimic natural tooth roots. This trend of patents can be seen as a limited local phenomenon in China, and in order to become a general trend, it is necessary to follow up on the flow of continuous research and patents and their application in clinical practice. In the past, research and development of dental implants focused on basic aspects in mechanical and biological aspects such as design and titanium surface modification, and recently, they further reflect clinical convenience of dentist by IT. In the future, with dental implant-related diseases increasing along with implant restoration, research and development will be more focused on methods or drugs for treating diseases and maintaining health for against already restored implants are expected to be more concentrated.

## Conclusion

In this study, we analyzed 32,237 patents filed from 2000 to 2020 through technical category evaluation by IPC analysis and text mining to understand the temporal and spatial progress of dental implant technology. Our results suggest that in the early 2000s, patented technologies concentrated on implant materials, structures, and surface treatment methods; however, recently filed patents related to dental implants are developing through convergence with IT/CT and focus mainly on improving the accuracy and convenience of diagnosis and treatment. These changes are being led by major countries in the rapidly growing Asian market rather than by existing technology powerhouses. Consequently, it is necessary to develop a new dental implant material technology that is highly applicable to the development of IT/CT hybrid technology and is suitable for a new manufacturing method. Additionally, institutes and companies participating in dental implant research must also change to adapt to the technological advancement trends that are in line with these market changes. Our study may provide important information to help basic and translational researchers and their financial supporters set their research directions to advance the development of dental implants.

## Data Availability

Not applicable.

## Abbreviations

IT: Information technology
CT: Communication technology
IPC: International patent classification
SLA: Sandblasting, large grit and acid etching
3Y-TZP: Yttrium-stabilized tetragonal zirconia
ATZ: Alumina-toughened zirconia
ZTA: Zirconia-toughened alumina
CAD/CAM: Computer-aided design/computer-aided manufacturing

## References

[1] National Institute on Aging (2011). Global health and aging, National Institute on Aging, National Institutes of Health.

[2] Butterworth C, McCaul L, Barclay C (2016). Restorative dentistry and oral rehabilitation: United Kingdom National Multidisciplinary Guidelines. J Laryngol Otol..

[3] United Nations. Department of Economic and Social Affairs. Population Division., World population ageing, 1950–2050, United Nations, New York 2002. https://www.un.org/development/desa/pd/sites/www.un.org.development.desa.pd/files/undesa_pd-2020_world_population_ageing_highlights.pdf.

[4] Sanderson WC, Scherbov S (2019). Prospective longevity: a new vision of population aging.

[5] Albrektsson T, Branemark PI, Hansson HA, Lindstrom J (1981). Osseointegrated titanium implants. Requirements for ensuring a long-lasting, direct bone-to-implant anchorage in man. Acta Orthop Scand.

[6] G. v. research, China Dental Implants Market Analysis - COVID19 - 2021-2027 - MedSuite - Includes: Dental Implants, Final Abutments & Surgical Guides, Grand view research, 2021, 104. https://www.researchandmarkets.com/reports/5314398/china-dental-implants-market-analysis-covid19#src-pos-2.

[7] Smith JA, Arshad Z, Trippe A, Collins GS, Brindley DA, Carr AJ (2018). The Reporting Items for Patent Landscapes statement. Nat Biotechnol.

[8] Mota F, Braga L, Rocha L, Cabral B (2020). 3D and 4D bioprinted human model patenting and the future of drug development. Nat Biotechnol.

[9] Higham KW, Governale M, Jaffe AB, Zulicke U (2019). Ex-ante measure of patent quality reveals intrinsic fitness for citation-network growth. Phys Rev E.

[10] Shalabi MM, Gortemaker A, Van't Hof MA, Jansen JA, Creugers NH (2006). Implant surface roughness and bone healing: a systematic review. J Dent Res..

[11] Wen CE, Yamada Y, Shimojima K, Chino Y, Asahina T, Mabuchi M (2002). Processing and mechanical properties of autogenous titanium implant materials. J Mater Sci Mater Med.

[12] Gahlert M, Burtscher D, Grunert I, Kniha H, Steinhauser E (2012). Failure analysis of fractured dental zirconia implants. Clin Oral Implants Res.

[13] Branemark PI (1983). Osseointegration and its experimental background. J Prosthet Dent.

[14] Section 101, United States Code, 2011 Edition, Supplement 5, Title 35 – PATENTS, USA. https://www.uspto.gov/web/offices/pac/mpep/s2104.html.

[15] Zhou X, Zhang Y, Porter AL, Guo Y, Zhu DH (2014). A patent analysis method to trace technology evolutionary pathways. Scientometrics.

[16] Albrektsson T, Zarb G, Worthington P, Eriksson AR (1986). The long-term efficacy of currently used dental implants: a review and proposed criteria of success. Int J Oral Maxillofac Implants.

[17] Han Q, Wang C, Chen H, Zhao X, Wang J (2019). Porous tantalum and titanium in orthopedics: a review. ACS Biomater Sci Eng.

[18] Guo Y, Xie K, Jiang W, Wang L, Li G, Zhao S, Wu W, Hao Y (2019). In vitro and in vivo study of 3d-printed porous tantalum scaffolds for repairing bone defects. ACS Biomater Sci Eng.

